# Advances in linear epitope-based subunit vaccines powered by artificial intelligence: current status and challenges

**DOI:** 10.3389/fimmu.2026.1902723

**Published:** 2026-09-07

**Authors:** Jingyu Quan, Lu Liu, Zijian Guo, Tiancheng Shan, Yuhua Shi, Xianbin Cheng

**Affiliations:** 1 Department of Thyroid Surgery, The Second Hospital of Jilin University, Jilin, China; 2 National Engineering Laboratory for AIDS Vaccine, School of Life Sciences, Jilin University, Jilin, China; 3 Department of Colorectal and Anal Surgery, The Second Hospital of Jilin University, Jilin, China

**Keywords:** HLA presentation, immunoinformatics, linear epitopes, peptide vaccines, precision vaccinology, vaccine delivery

## Abstract

In this review, linear epitope-based subunit vaccines are systematically profiled to outline their current developmental status, integrating recent advances in computational immunogenic epitope screening, site-specific chemical modification for stability enhancement, next-generation delivery platform matching, and ongoing clinical translation efforts. Deep learning-based MHC-binding prediction tools including NetMHCpan, MHCflurry 2.0, and MARIA, supported by robust empirical evidence, are rigorously evaluated, with notable improvements observed in the efficiency of identifying B/T cell linear epitopes with high immunogenicity, while it is also noted that the field still faces core bottlenecks such as weak *in vivo* immunogenicity, rapid enzymatic degradation, and insufficient cross-protection against viral variants. According to this integrative evidence-based assessment, feasible optimization pathways are further outlined, including the synergy between AI-driven epitope structural design and virus-like particle-based delivery systems, to provide practical references for the rational development of next-generation broad-spectrum linear epitope-based vaccines.

## 1 Introduction

Vaccines are essential tools for infectious disease prevention and control, representing a highly effective public health intervention (1). Their core mechanism relies on mimicking pathogen-derived antigenic signals to recapitulate natural infection, thereby activating host immune defenses, stimulating antigen-specific adaptive immunity, and establishing long-term immunological memory. Conventional vaccines, including inactivated and live attenuated formulations, have played a critical role in historical disease control. However, limitations such as variable development timelines, high platform-dependent manufacturing costs, and pathogen-dependent biosafety requirements have driven the exploration of next-generation vaccine platforms (2, 3). Against this backdrop, subunit vaccines composed of defined linear epitope sequences (epitope peptides) have emerged as a promising alternative platform.

Epitopes are specific antigenic regions recognized by T or B lymphocytes: T cell epitopes are typically short peptides presented by major histocompatibility complex (MHC) molecules, while B cell epitopes are divided into linear and conformational subtypes (4). These epitopes can be chemically synthesized, with advantages including low molecular weight, well-defined composition, and high safety (5, 6).

As potential next-generation vaccine candidates, linear epitope-based subunit vaccines can elicit antigen-specific cellular and humoral immunity for targeted protection, and avoid the inherent drawbacks of conventional vaccines by containing no exogenous genetic material, with improved safety profiles (7, 8). Current research in this field covers infectious disease prevention, cancer immunotherapy, and autoimmune disease modulation, demonstrating broad translational potential (6, 7).

Characterized by well-defined antigenic components, favorable safety, and adaptability to rapid antigen sequence updates, linear epitope-based subunit vaccines are a promising platform for responding to emerging infectious disease outbreaks and advancing precision vaccinology. However, their end-to-end R&D pipeline faces three core bottlenecks: (1) low efficiency and accuracy of linear epitope identification, (2) inherent low immunogenicity of short linear peptides, and (3) insufficient broad-spectrum protective efficacy against rapidly evolving pathogens. Integrated AI and cross-disciplinary solutions are being explored to address these challenges, fueling the R&D of linear epitope vaccines (9).

The first core bottleneck is the low efficiency and accuracy of linear epitope identification: conventional computational or experimental methods often fail to meet the dual requirements of high sequence conservation and robust immunogenicity. Given the rapidly evolving pathogen mutations and extensive global human leukocyte antigen (HLA) allele polymorphism, traditional approaches have obvious shortcomings in throughput and accuracy, often missing valuable conserved epitopes or selecting epitopes that cannot elicit immune responses in diverse populations (10, 11). AI, especially deep learning architectures, offers targeted solutions to this limitation (12). For instance, ConFormer, a hybrid model integrating convolutional operations and self-attention mechanisms, can accurately decode HLA-epitope binding interaction patterns, significantly improving the precision of epitope-HLA binding prediction (13). Additionally, AI enables high-throughput mining of conserved epitopes across diverse pathogenic strain proteomes, with algorithms optimized to accommodate global HLA allele polymorphism (14).

The second core bottleneck is the inherent low immunogenicity of short linear peptides: their high structural flexibility makes them prone to enzymatic degradation, and they cannot effectively trigger antigen presentation and T cell response pathways. They usually require adjuvants or delivery carriers for effective immune activation, but the targeting capability and stability of existing peptide delivery systems still need improvement, further raising R&D costs and clinical translation risks (15, 16). To address this, AI optimization strategies under the “physics-data fusion” paradigm have made preliminary progress: for example, Free Energy Perturbation-assisted Machine Learning (FEPaML) integrates physics-based molecular simulations with data-driven model training to precisely modify epitope structural conformations, enhancing their binding affinity for key immune molecules such as MHC and T cell receptors (17). In addition, AI supports the rational design of multi-epitope vaccines by screening and prioritizing peptides with high immunogenic potential, assembling optimized epitope repertoires that strongly potentiate antigen-specific T cell activation (18).

The third core bottleneck is insufficient broad-spectrum protective efficacy: continuous pathogen evolution drives epitope sequence drift, and traditional vaccine platforms, with long R&D cycles and slow update iterations, cannot quickly adapt to emerging variants, posing immune escape risks (19). To address this, AI’s high-throughput data processing capacity enables rapid mining of massive sequence datasets from mutated strains, facilitating the identification of functionally conserved viral regions with low mutation rates to mitigate immune escape risks (20). Additionally, AI models can be iteratively retrained and refined using genomic and immunological data from new variants, enabling the rational design of broad-spectrum vaccines with cross-reactivity against multiple mutant strains (21).

The retrieval strategy employed systematic searches of PubMed, Web of Science, and Scopus (January 2000–December 2025, English-language) using the combined keywords: “linear epitope vaccine”, “peptide vaccine”, “subunit vaccine”, “epitope prediction”, “artificial intelligence”, “machine learning”, “deep learning”, “MHC binding”, “HLA”, “immunoinformatics”, “reverse vaccinology”, “nanoparticle delivery”, “virus-like particle”, and “clinical trial”. Additional records were obtained via citation tracking of key reviews and primary studies; clinical-trial data were cross-checked against ClinicalTrials.gov and the WHO ICTRP (cut-off 31 Dec 2025). All computational methods cited were grouped into five categories: (i) conventional bioinformatics (sequence alignment, homology search); (ii) machine learning (SVM, random forest); (iii) deep learning (ANN, transformer, protein language models); (iv) molecular simulation (MD, docking); and (5) physics-based methods (FEP calculations).

“Linear epitope-based subunit vaccines” are defined as those whose antigenic component consists of chemically synthesized or recombinantly expressed linear peptide sequences representing discrete epitope regions. This includes: (i) short linear peptides (8-15 aa for MHC-I, 15-30 aa for MHC-II); (ii) synthetic long peptides (SLPs, 20-50 aa); and (iii) concatenated multi-epitope polypeptides. Peptide-carrier conjugates and VLPs displaying linear peptide epitopes as the primary antigen are included; full-length recombinant proteins, conventional VLPs from intact viral proteins, and mRNA vaccines encoding native full-length antigens are excluded unless their use for linear epitope design is explicitly justified. Related technologies are discussed only insofar as they provide methodological insight or comparative benchmarks. Although peptide vaccines generally possess a favorable safety profile due to their defined, homogeneous composition, off-target immunogenicity, molecular mimicry-autoimmunity, and adjuvant toxicity remain case-specific risk-benefit considerations.

This review evaluates the design rationale, advantages, and current frontiers of linear epitope-based subunit vaccines, underscores their relevance to public-health emergency response, and delineates how immunoinformatics, bioinformatics, and allied disciplines accelerate the full R&D pipeline. AI-driven deep learning models are anticipated to enhance target discovery speed and structural optimization. Persistent challenges include suboptimal peptide delivery systems, pathogen mutation-driven efficacy loss, and technical bottlenecks in AI integration for epitope vaccine development; resolving these demands cross-disciplinary innovation. Future progress will likely stem from the convergence of single-cell multi-omics, digital-twin modeling, and targeted nanoparticle delivery, advancing precision vaccinology and AI-enabled delivery toward a more proactive, pre-emptive disease-prevention paradigm.

## 2 AI-assisted epitope sequence prediction and vaccine development

### 2.1 Epitope sequence prediction and screening

Integrating immunoinformatics, computational biology, and AI enables systematic extraction of immunogenic, conserved epitopes from viral, bacterial, and tumor-specific antigens, as well as high-throughput screening of large-scale epitope libraries to identify viable candidates (4). This integrated approach markedly boosts screening efficiency and accuracy, while circumventing the labor-intensive conventional wet-lab workflows for epitope mapping (22, 23). Epitope-HLA binding affinity assessment leverages curated public resources: the Protein Data Bank (PDB), which archives large volumes of annotated peptide-MHC (pMHC) crystal structure data, and the Immune Epitope Database (IEDB), which aggregates extensive experimentally validated epitope binding affinity datasets (24). Widely used algorithms including NetMHCpan (25) and MHCflurry 2.0 (26) enable high-throughput quantification of binding affinities; a sub-50 nM IC50 value is the conventional cutoff for strong binding potential in most assay systems, though this threshold varies across HLA alleles, experimental platforms, and MHC classes, with percentile ranks generally preferred for cross-allele comparability. Critically, predicted binding affinity is not equivalent to immunogenicity: additional biological factors—including intracellular antigen processing, T-cell receptor (TCR) repertoire diversity, and central/peripheral immune tolerance—ultimately determine whether a presented peptide can elicit a functional antigen-specific T-cell response (27).

Benchmarking on IEDB datasets reveals notable performance differences among leading MHC-binding predictors: NetMHCpan 4.1 achieves a median AUC of 0.92–0.95 across diverse HLA alleles, while MHCflurry 2.0 delivers comparable performance (AUC 0.90–0.94) with faster inference speeds (26). These metrics, however, are derived primarily from peptide elution datasets that overrepresent common HLA alleles (e.g., *HLA-A02:01, HLA-A24:02*) (28), resulting in systematically reduced predictive accuracy for rare alleles with limited training data (25). Performance gaps are even more pronounced for HLA class II prediction: while MARIA reaches an AUC of 0.89–0.92 on held-out datasets, it exhibits substantial allele-specific variation, with scores ranging from 0.76 to 0.94 across HLA-DR alleles (29). A universal limitation of all existing platforms is their inability to distinguish MHC-binding peptides from those that are actually intracellularly processed and surface-presented—a distinction critical for vaccine design that cannot be captured by binding affinity alone. Additionally, most tools show poor performance on non-canonical peptide lengths and post-translationally modified peptides, both of which are prevalent in tumor antigens but underrepresented in training cohorts.

The improving accuracy of HLA class II presentation prediction has propelled the design of broadly protective universal vaccines to the forefront of vaccine research. By integrating global HLA-A/B/C allele frequency data (30) with computational epitope conservation screening, vaccine universality can be substantially enhanced (22, 23).

Reverse vaccinology approaches have demonstrated robust efficacy in vaccine design against diverse pathogens. For example, studies targeting HPV16 E6/E7 oncoproteins leveraged PaVE database sequence data, integrating bioinformatic prediction with experimental validation to identify multiple immunogenic cytotoxic T lymphocyte (CTL) epitopes (31), providing a foundational framework for therapeutic HPV vaccine development. Leveraging immunoinformatics, researchers have also computationally designed multi-epitope vaccines against a range of pathogens including Helicobacter pylori (32), HPV (33), and enterotoxigenic *Escherichia coli.*

Epitopes are classified into B-cell and T-cell subsets based on their recognition profiles and structural features, a distinction with profound implications for vaccine design. B-cell epitopes are directly recognized by B cells to trigger humoral immunity and antigen-specific antibody production. A key limitation of linear B-cell epitopes is their inability to recapitulate the native conformation, accessibility, and physicochemical context of the corresponding epitope region in the intact antigen; as a result, antibodies raised against isolated linear peptides often fail to recognize native pathogen or tumor antigens, requiring rigorous structural validation.

**Figure 1:**
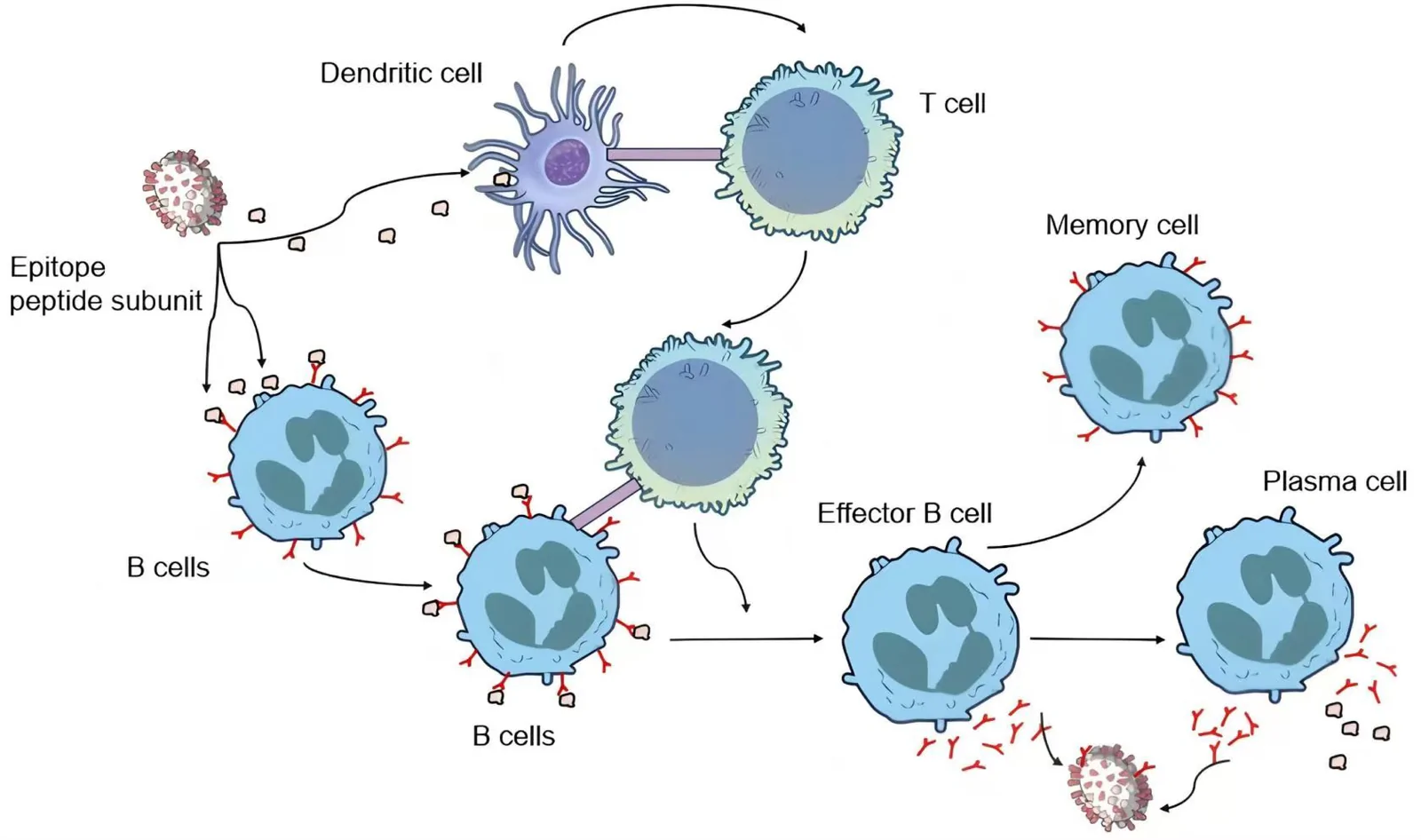
A schematic diagram of the immune response process involving B cells and T cells binding with epitope peptides. Figure created with Microsoft Office PowerPoint. The diagram should distinguish between short peptides, synthetic long peptides, and carrier-conjugated peptide vaccines, and should illustrate peptide/peptide-carrier uptake by dendritic cells, antigen processing, MHC class.

In contrast, T-cell epitopes require intracellular processing, loading onto MHC class I/II molecules, and surface presentation to engage TCRs and drive cell-mediated immune responses (34), including the activation of helper T cells (Th) that provide cognate support to B cells via T-follicular helper (Tfh) interactions rather than nonspecific cytokine release. Design of T-cell epitopes depends on a distinct set of parameters: antigen processing, peptide generation and transport, MHC loading efficiency, peptide-MHC complex stability, HLA restriction, TCR repertoire diversity, immunodominance, and immune tolerance/exhaustion. These requirements must be fully accounted for in sequence optimization, chemical stabilization, and structural prediction workflows (see **Figure 1**).

T-cell epitope selection is guided by sequence, length, and structural attributes, with HLA binding affinity serving as a primary initial screening criterion (35). Widely used prediction tools include IEDB-recommended NetMHCpan and NetMHCIIpan platforms (leveraging the NNAlign_MA model) (25, 36) and HLA class I-specific MHCflurry 2.0 (26). Robust candidate prioritization requires integrated assessment of immunogenicity, sequence conservation, and global population HLA coverage (37, 38).

### 2.2 Development of linear epitope subunit vaccines

To enhance the stability and immunogenicity of linear epitopes and overcome limitations of native epitopes (e.g., proteolytic vulnerability and suboptimal immunogenicity), diverse optimization strategies have been developed, with AI emerging as a core enabling technology.

1. Chemical Modification Strategies, including glycosylation and phosphorylation, are the most widely used chemical modification approaches for linear epitopes, both of which can substantially improve epitope stability and immunogenicity. It is critical to distinguish direct modifications to peptide epitopes from modifications applied to adjuvants or carrier moieties, which fall under separate formulation optimization categories.

Sialylation, entails covalent attachment of sialic acid residues via α-2,6-glycosidic linkages to peptide epitopes. This modification blocks recognition and hepatic clearance by sialic acid-binding glycoprotein receptors, and suppresses DC-SIGN receptor-mediated endocytosis (39), extending the circulating half-life of modified epitopes from 2 h to 12 h. Preclinical studies of sialylation-modified NY-ESO-1 epitope vaccines report a ~4-fold increase in tumor-infiltrating T lymphocytes (TILs) relative to unmodified peptide controls. That said, this finding is derived exclusively from a murine B16 melanoma model; its reproducibility across independent laboratories and tumor types has not been validated, and interpretation must account for the specific experimental parameters (peptide dose, administration route, tumor model) underpinning the observed effect.

Sialylation exerts context-dependent dual effects on vaccine performance: while it improves pharmacokinetic properties, cancer-associated aberrant sialylation can mask tumor antigen epitopes, establish an immunosuppressive microenvironment, and facilitate tumor immune escape (40). Targeting tumor-derived sialylated glycoproteins is a promising therapeutic strategy, as it can inhibit tumor metastasis, unmask hidden antigen epitopes, and amplify anti-tumor immune responses. Further characterization of tumor-specific sialylation patterns will help identify novel glycan targets and guide the development of personalized cancer immunotherapies (39). Overall, the impact of sialylation on vaccine immunogenicity is not universally beneficial, and varies with construct design, glycosidic linkage type, cellular receptor engagement profile, and experimental context.

The integration of AI-driven predictive models and large-scale curated immunogenicity datasets has streamlined epitope modification workflows and accelerated vaccine development timelines (19). Unlike traditional epitope modification workflows that rely on labor-intensive empirical trial and error, AI enables direct prediction of optimal modification strategies via dedicated modification-immunogenicity prediction models (41). When provided with linear epitope sequences and candidate modification sites, AI can forecast alterations in peptide stability and immunogenic potency of modified segments, eliminating low-yield or futile modification trials (42). Additionally, AI supports high-throughput screening of modification sites that maximize peptide half-life and T-cell activation efficiency, making this computational-first approach a cost-effective strategy to improve candidate molecule development efficiency.

Phosphorylation modification, another widely adopted chemical modification strategy, directly targets peptide antigens, and must be distinguished from adjuvant-related modifications such as the phosphorothioate backbone of CpG oligodeoxynucleotides (CpG-ODN), which are covered in the adjuvant optimization section. Preclinical work from the MD Anderson Cancer Center demonstrates that epitope phosphorylation can reinvigorate suppressed immune responses, representing a promising approach to overcome tumor-induced immune tolerance (43).

2. Nanoparticle Delivery Systems serve as effective carriers to enhance epitope delivery efficiency and immunogenicity. Fabricated by encapsulation, conjugation, or related technologies, NP synthesis is multifunctional, requires low purification standards, and is readily scalable for manufacturing (44). NPs preferentially target antigen-presenting cells (APCs), possess a high surface-area-to-volume ratio, load cargo efficiently, and are amenable to surface modification. These properties protect antigens from premature degradation and markedly improve immune responses, thereby increasing vaccine efficacy (44).

*Chitosan* – a cationic biopolymer with mucosal adhesivity, antimicrobial activity, low allergenicity, and biocompatibility – has been extensively studied as a vaccine adjuvant (45). It exerts its adjuvant effect primarily through activation of the cGAS-STING pathway, driving robust Th1-skewed immunity essential for protection against infections such as tuberculosis (46). Optimized NPs, defined by size, charge, hydrophobicity, and surface-targeting ligands, can more effectively shuttle conjugated antigens to APCs, eliciting durable immune responses (47).

Dendritic polymers and lipid nanoparticles (LNPs) also show promise for preventing peptide degradation. For instance, fourth-generation polyamide-amine (PAMAM G4) dendrimers offer 64 potential surface modification sites and high epitope-loading capacity (48). Note that many PAMAM studies focus on DNA vaccine delivery; their relevance to peptide vaccines must be justified explicitly. G5 PAMAM conjugated with MHC class II-targeting peptides can selectively deliver DNA to APCs *in vivo*, transfect dendritic cells, induce high-affinity T-cell responses and antitumor immunity, thus providing an ideal platform for DNA vaccine development (49). For peptide-based vaccines, NP performance should be judged by peptide encapsulation efficiency, surface conjugation, release kinetics, lymphatic trafficking, and APC uptake — not extrapolated from nucleic-acid delivery data.

NP carrier design must match the physicochemical traits of the peptide (charge, hydrophobicity) (50). Advances in AI now enable “peptide-carrier” intelligent matching (51). By training on extensive nanoparticle delivery datasets, AI can predict key parameters — encapsulation efficiency, targeting ability, and solubilization-release kinetics — for a given epitope peptide, allowing direct selection of the optimal NP formulation, reducing development costs and accelerating timelines.

3. Virus-Like Particle (VLP) Technology are self-assembled arrays of viral structural proteins that mimic native virus morphology and size while lacking genetic material, rendering them non-infectious (52, 53). They can be produced in diverse cell-based or cell-free expression systems (45, 54), facilitating reconstruction of viral architectures. This approach is gaining traction in preventive medicine, with numerous VLP-based vaccine candidates against various infectious agents under development (45).

A critical distinction exists between native VLP vaccines (e.g., HPV and HBV vaccines derived from full-length L1 or HBsAg proteins) and heterologous VLP platforms engineered to display inserted linear peptide epitopes. The latter are directly relevant to this review as epitope display vehicles; the former fall outside the linear peptide vaccine definition except insofar as they inform epitope-presentation strategies. For example, codon-optimized HPV16 L1 cloned into pFastBac, expressed in Sf9 insect cells via the Bac-to-Bac system, purified by Ni-NTA chromatography, verified by SDS-PAGE/Western blot, and confirmed by transmission electron microscopy to form intact VLPs, demonstrates that the baculovirus/Sf9 system can produce laboratory-scale assembled VLPs (55). However, laboratory-scale expression alone does not guarantee industrial suitability; scalability demands additional evidence on yield, purification consistency, batch-to-batch reproducibility, long-term stability, and GMP controls.

Beyond prophylactic vaccines, VLPs are exploited for targeted drug delivery. Their virus-like size and shape confer strong permeability and retention effects, making them ideal carriers for delivering therapeutics to tumor tissue for treatment and imaging (56). The absence of viral genomes eliminates replication risk in target cells, enhancing safety for immunocompromised or elderly recipients (52).

Effective VLP technology requires precise surface display of linear epitopes. AI can overcome assembly and low-immunogenicity challenges by employing structural prediction tools (e.g., AlphaFold) to model interactions between linear epitopes and VLP capsid proteins (57). AI may forecast post-insertion structural stability and epitope exposure, thereby avoiding insufficient presentation that could blunt immune activation. Rapid AI-driven screening of fusion strategies that stabilize assembly and maximize epitope display mitigates risks of VLP assembly failure or reduced immunogenicity.

4. Amino Acid Sequence Optimization is critical for enhancing the efficacy of linear epitope-based subunit vaccines. To improve peptide stability, protease-susceptible L-amino acids can be substituted with D-amino acids (58), or non-natural moieties such as N-methylated amino acids can be incorporated to evade protease recognition, thereby preventing *in vivo* degradation of the vaccine construct. N-terminal acetylation or C-terminal amidation blocks exopeptidase cleavage sites to extend vaccine half-life (59). However, these protease-resistance-enhancing modifications may concurrently impair antigen processing, MHC binding, or T-cell receptor (TCR) recognition, and such trade-offs must be explicitly accounted for in vaccine design.

To augment immunogenicity and delivery efficiency, disulfide bond integration (60) or cyclic structure engineering (61) can be used to stabilize peptide conformation. Phage display technology can also be applied to screen high-affinity mutants that enhance neutralizing antibody recognition (62). Modulating key residues in the MHC binding groove improves peptide-MHC complex stability, while multiepitope conjugation broadens the immune response spectrum. Additionally, tuning amino acid polarity and hydrophobicity optimizes peptide solubility and delivery performance.

As epitope immunogenicity is intrinsically determined by amino acid sequence, sequence screening and optimization are indispensable steps in vaccine design. AI can mine large-scale datasets to identify potent peptides leveraging immunogenicity databases such as the IEDB (63). It can predict beneficial single- or multi-point mutations that enhance T-cell and B-cell recognition, while confirming the non-toxicity of optimized sequences. AI can also assess peptide solubility and protease resistance to ensure that final optimized candidates are not only highly immunogenic, but also amenable to scalable production and *in vivo* delivery.

**Table 1** summarizes the key applications of AI and computational methods in peptide vaccine development. When evaluating these tools, it is critical to distinguish computational predictive performance from prospective experimental performance: a model that accurately predicts peptide binding does not necessarily demonstrate the ability to identify protective vaccine antigens.

**Table 1:** Applications of AI and computational methods in peptide vaccine development.

Applications	Specific task	AI/computational methods	Key results/performance	Limitations
Epitope Prediction	Predict HLA-binding affinity	NetMHCpan, MHCflurry 2.0, MARIA	MARIA achieved an AUC of 0.89–0.92	Highly dependent on high-quality training data
Sequence Screening	Identify conserved and immunogenic epitopes	Deep learning, immunoinformatics	Rapid analysis of multiple strains and HLA alleles	Rare mutations may be overlooked
Structure Optimization	Predict modification effects and structural stability	ConFormer, FEPaML, AlphaFold2	Improved affinity, stability, and epitope exposure	Difficult to simulate dynamic conformational changes
Delivery Design	Optimize “peptide–carrier” matching	Machine learning based on nanoparticle datasets	Predicted encapsulation efficiency, targeting ability, and release rate	Requires large amounts of high-quality data
Preclinical Evaluation	Virtual immune screening and efficacy prediction	Molecular dynamics simulation, systems biology	Reduced cost and development time	Experimental validation is still necessary
Mutation Response	Track viral evolution and predict immune escape	Deep learning models	Helped design broad-spectrum vaccines (e.g., Omicron-related studies)	Predictions may lag behind newly emerging variants

## 3 Advances in subunit vaccine research based on epitope sequences

As immunology and biotechnology converge, linear epitope-based subunit vaccines have emerged as a transformative vaccination modality. By enabling precise antigen design and potent immune activation, they overcome key limitations of traditional vaccines. Their applications now extend far beyond infectious disease prophylaxis to cancer immunotherapy, autoimmune disease intervention, and animal health improvement, redefining the conventional paradigm of vaccinology.

### 3.1 Vaccines against infectious diseases

In malaria vaccine research, epitope peptides targeting specific Plasmodium parasite proteins have been shown to effectively trigger immune responses and bolster host immunity (64). Novel poly(lactic acid) (PLA) nanoparticle formulations incorporating PfEMP1 ligands, immunopeptides, and linkers have emerged as promising candidates for preventing complex malaria infection (note: the “PLCs” abbreviation in the cited source may be a typographical error requiring verification against the original study).

The RTS,S/AS01 malaria vaccine, developed by GlaxoSmithKline based on circumsporozoite protein (CSP) epitopes, is a recombinant protein particle vaccine (composed of CSP fused to hepatitis B surface antigen) rather than a chemically synthesized linear peptide vaccine. It is cited here to illustrate epitope-targeted design principles relevant to peptide vaccine development, not as a direct example of linear epitope peptide vaccines. In a large Phase III clinical trial, this vaccine demonstrated moderate protective efficacy in African children: 55.8% (95% CI: 44.4–64.1) against clinical malaria and 47.3% (95% CI: 27.5–61.7) against severe malaria at 12 months post a three-dose regimen in children aged 5–17 months (65). However, protective efficacy wanes over time, and is significantly reduced in high-transmission settings and in infants aged 6–12 weeks (66). Owing to its favorable risk-benefit profile, the World Health Organization (WHO) recommended its widespread use in 2021 for children living in high-transmission malaria regions, including sub-Saharan Africa, marking a milestone for both malaria control and broader parasitic disease prevention (67).

During COVID-19 vaccine development, multiple vaccines using SARS-CoV-2 surface protein (e.g., spike protein) epitopes as immunogens achieved favorable immunological outcomes (68). Notably, Moderna’s mRNA-1273, despite its high efficacy, is an mRNA vaccine encoding a full-length stabilized spike protein rather than a linear epitope peptide vaccine. It is discussed here only to highlight how epitope region insights have informed broader vaccine strategies, and should not be classified as a linear epitope-based subunit vaccine. The TENT5A-mediated poly(A) tail extension in macrophages, which enhances mRNA stability and antigen protein expression to augment overall vaccine immunogenicity (69), is an mRNA-specific mechanism that differs fundamentally from the biological basis of peptide vaccines.

Additionally, a broad-spectrum coronavirus vaccine developed by the Chinese Academy of Sciences, targeting the conserved receptor-binding domain (RBD) region, has demonstrated cross-neutralizing antibody titers ≥1:4096 against both SARS-CoV-2 and MERS-CoV (70), though further clinical trials are required for validation.

The integration of AI has markedly improved the efficiency and adaptability of linear epitope vaccine research against infectious diseases. To address the challenge of waning vaccine efficacy driven by viral mutations and associated immune escape risks, researchers have combined deep learning with viral mutation analysis to predict immune escape risk. For instance, in SARS-CoV-2-focused studies, AI-designed multi-epitope constructs are predicted to activate both CD8+ and CD4+ T-cell responses, and computational analyses indicate potent neutralizing activity against numerous variants (71). It is critical to emphasize that these remain in silico predictions pending validation by cellular or animal experiments: computational immune simulations are hypothesis-generating tools, and cannot be regarded as experimental evidence of immunogenicity.

In tuberculosis vaccine development, AI is applied to optimize epitope arrangement and linker peptide design (72), thereby enhancing the structural stability and immunogenicity of chimeric antigens (73).

### 3.2 Vaccines against cancer

The concept of vaccines has expanded beyond their traditional role in preventing infectious diseases to include cancer prophylaxis and therapy. As a modality of therapeutic cancer vaccine, linear epitope subunit vaccines remain primarily in the research and development (R&D) phase, facing multiple barriers that limit their broad clinical application. Discussions of cancer vaccines must distinguish between prophylactic vaccines (e.g., human papillomavirus (HPV) prophylactic vaccines) and therapeutic vaccines (e.g., neoantigen-based personalized vaccines), as their mechanisms, target populations, and development pathways differ substantially.

It should be clarified that ^177^Lu-dotatate, though a peptide-based therapeutic, is classified as peptide receptor radionuclide therapy (PRRT) rather than a cancer vaccine: its mechanism relies on targeted delivery of ionizing radiation to tumor cells expressing somatostatin receptors, rather than immune activation. Thus, this agent is not an appropriate example of cancer vaccine progress and should be excluded from vaccine-related discussions, even though the FDA-approved ^177^Lu-dotatate effectively treats gastroenteropancreatic neuroendocrine tumors via conjugation of radioactive isotopes to targeted peptides (74).

For instance, to address HPV16-associated cervical cancer, researchers have screened multi-epitope peptides derived from HPV16 E5, E6, and E7 proteins to construct HBc-E765m chimeric virus-like particles (cVLPs) (7). In murine models, this vaccine induced robust cytotoxic T lymphocyte (CTL) responses and serum IgG antibody production, effectively suppressed tumor growth, and shows promise as an immunotherapeutic option for HPV16-related cervical intraepithelial neoplasia (CIN) and cervical cancer (75).

Caution is required when comparing therapeutic cancer vaccines to conventional treatments. While these vaccines generally exhibit a more favorable safety profile than many cytotoxic agents, their safety is highly context-dependent, varying with the target antigen, adjuvant, delivery platform, combination regimen, dose, and patient population. Claims that cancer vaccines are broadly safer than radiotherapy, chemotherapy, and endocrine therapy oversimplify the complex risk-benefit landscape; such comparisons must explicitly reference specific vaccine candidates and clinical contexts (76).

AI has emerged as a pivotal tool for overcoming barriers to the development of linear epitope vaccines for low tumor mutational burden (low-TMB) cancers (77). Traditional methods often fail to identify sufficient neoantigens in these malignancies, while AI can integrate multidimensional sequencing data to uncover cryptic antigen sources (78). Additionally, AI streamlines the entire vaccine design pipeline by improving the accuracy of neoantigen prediction, immunogenicity assessment, and vaccine structural design (79). Experimental data show that vaccines constructed using AI-selected epitopes significantly increase CD8^+^ and CD4^+^ T-cell infiltration in tumor tissues, promote interferon (IFN)-γ secretion, and further amplify anti-tumor immune responses when combined with PD-1 inhibitors, thereby effectively suppressing tumor growth and prolonging patient survival (80).

### 3.3 Other applications

Research on linear epitope subunit vaccines is also advancing in fields including autoimmune diseases and allergic disorders. While some studies remain at the exploratory stage, they provide novel insights and directions for the prevention and treatment of these conditions (81). In the autoimmune disease space, the GAD65 epitope vaccine has shown promise in delaying the onset of islet inflammation in newly diagnosed type 1 diabetes patients in early-phase clinical trials (82), offering new perspectives for type 1 diabetes management. Additionally, linear epitope subunit vaccines have demonstrated substantial potential in veterinary applications, effectively preventing and controlling major animal diseases such as porcine epidemic diarrhea virus and classical swine fever virus (83).

## 4 Interdisciplinary approaches supporting linear epitope subunit vaccine design

The convergence of immunoinformatics, bioinformatics, and allied disciplines provides critical technological support for epitope peptide identification, screening, and vaccine design optimization.

### 4.1 Immunoinformatics enhancing epitope prediction accuracy

Immunoinformatics (also called computational immunology) is an interdisciplinary field integrating computer science and experimental immunology. It leverages computational approaches to analyze large-scale immunological datasets, providing mechanistic insights into immune response regulation (36). With advances in computing technology, computer-aided vaccine design has become a core tool for developing linear epitope subunit vaccines: by predicting linear epitopes with immunostimulatory potential, researchers can rapidly screen candidate sequences and optimize vaccine formulations (84).

Deployment of immunoinformatics tools, combined with integration of multi-omics data from protein structure repositories (e.g., Protein Data Bank, PDB) and gene expression databases (e.g., Gene Expression Omnibus, GEO), enables the construction of predictive models for epitope-immune response interactions, improving screening accuracy (85). For instance, DeepMind’s AlphaFold2 AI system has predicted nearly the entire structure of the human proteome and released its database for public use (86). It should be noted that AlphaFold2 only generates structural predictions and does not directly model antigen processing, HLA (human leukocyte antigen) presentation, TCR (T cell receptor) recognition, antibody elicitation, or protective immunity. Nonetheless, this landmark resource provides an unprecedented structural foundation for rational epitope design, which may accelerate the development of novel vaccines.

### 4.2 Bioinformatics accelerating novel vaccine development

Bioinformatics plays an indispensable role in vaccine design, encompassing subfields such as immunoinformatics and reverse vaccinology. It significantly shortens vaccine development cycles and reduces costs associated with laboratory-based characterization of pathogen gene products (87). Conventional experimental methods often require lengthy pathogen culture and protein extraction workflows; even for rapidly growing pathogens, large-scale protein testing remains costly and time-intensive. Advances in immunoinformatics algorithms and databases now enable rapid analysis of sequence regions and identification of potential immune-binding sites (85), accelerating new vaccine development and substantially improving research efficiency.

## 5 Challenges and development trends in linear epitope subunit vaccines

Following comprehensive analysis of AI applications, delivery platforms, and clinical progress in preceding sections, it is critical to examine the barriers currently constraining the translational potential of linear epitope subunit vaccines. These challenges span immunological hurdles (immune tolerance, peptide instability), pathogen-driven immune escape, and technical limitations of AI in modeling dynamic protein conformations and predicting functional immune outcomes. Addressing these bottlenecks requires both technological innovation and a systematic understanding of how each obstacle can be leveraged to drive future advancement (see **Table 2**).

**Table 2:** Key challenges and future directions in linear epitope subunit vaccine development.

Challenge category	Specific problem description	Example in the text	Future development trend
Immunological challenge	Immune tolerance	WT1 epitope tolerated in healthy individuals	Overcoming/bypassing immune tolerance
Delivery bottleneck	Short half-life of free peptides (<2 h)	Need for carrier protection	Precision delivery
Pathogen variation	Immune escape	Omicron spike protein mutations	Broad-spectrum vaccines
AI technical challenge	Difficulty in simulating dynamic conformations	Gap between static prediction and functional validation	Systematic immune prediction
Translational gap	Antibodies fail to recognize native virus	Optimized antibody still ineffective at 100 μg/mL	Functional/mechanistic understanding

### 5.1 Key challenges

A core immunological barrier is immune tolerance, which impairs antigen recognition and dampens protective immune responses, reducing overall vaccine efficacy (88). The implications of tolerance vary by indication: it is undesirable for infectious disease vaccines, must be selectively broken for cancer vaccines targeting self-like tumor-associated antigens (with inherent autoimmunity risk), and is the intended therapeutic goal for vaccines against autoimmune and allergic diseases. These context-dependent requirements must inform tolerance-related design strategies. For instance, the WT1 tumor epitope exhibits natural immune tolerance in healthy individuals (89), making tolerance circumvention a key priority for oncology epitope vaccines.

Compounding this, many linear epitopes are rapidly degraded *in vivo*, with serum half-lives often below 2 hours, curtailing their duration of action and immunogenicity (88). The short lifespan of free linear epitopes necessitates robust delivery systems to enhance their immunogenicity and targeted delivery to immune cells (90). While optimized delivery platforms can significantly improve epitope uptake and reduce off-target exposure, current vectors still face persistent limitations in safety, efficacy, and tissue targeting that require further resolution (88).

Another major barrier is immune escape driven by high pathogen mutation rates, which alter epitope sequences and erode vaccine breadth and durability (91). The emergence of SARS-CoV-2 Omicron subvariants (particularly BA.5) exemplifies this challenge: spike protein mutations abrogated neutralization by antibodies induced against early strain epitopes, leading to a marked decline in the efficacy of first-generation vaccines against symptomatic infection (92, 93). This has driven interest in broad-spectrum coronavirus vaccines, with rationally designed linear epitope subunit vaccines targeting conserved viral regions emerging as a leading strategy to overcome ongoing viral evolution.

While AI has shown promise in predicting and designing immunogenic linear epitopes, it faces distinct interdisciplinary and translational barriers:

1. Immune tolerance barrier A subset of epitope subunit vaccines are designed to modulate immune responses against self-antigens, particularly for cancer and autoimmune disease therapies (94). Certain tumor-associated epitopes, such as the WT1 epitope, exhibit natural immune tolerance in healthy individuals (89). Overcoming or bypassing this tolerance thus remains a core challenge in linear epitope subunit vaccine development. The therapeutic objective varies substantially by indication: breaking tolerance is required for cancer vaccines targeting self-like tumor antigens, but carries inherent autoimmunity risk; inducing tolerance is the goal for autoimmune and allergy vaccines, yet may compromise protective immunity against pathogens.

2. Delivery efficiency limitations Unformulated linear epitopes typically have a serum half-life of less than 2 hours, necessitating robust delivery systems to enhance their immunogenicity (90). An optimized delivery system can significantly improve the efficiency of linear epitope delivery to target immune cells while reducing off-target exposure. However, current delivery vectors still face limitations in safety, immunogenicity, and tissue targeting, posing unresolved challenges for clinical translation (88).

3. Pathogen mutation -driven immune escape High mutation rates of pathogens alter native epitope sequences or generate novel immunodominant epitopes, eroding the breadth and durability of existing linear epitope subunit vaccines and impeding immune recognition of original vaccine-matched epitopes (90). The emergence of SARS-CoV-2 Omicron subvariants, particularly BA.5, exemplifies this challenge: spike protein mutations drove immune escape from many neutralizing epitopes identified based on earlier strains, severely reducing the protective efficacy of first-generation vaccines against symptomatic infection (92, 93). This scenario has spurred development of broad-spectrum coronavirus vaccines, with rationally designed linear epitope subunit vaccines targeting conserved viral regions emerging as a pivotal strategy to counter ongoing viral evolution.

4. AI integration and validation challenges

AI faces substantial interdisciplinary hurdles in linear epitope vaccine development. While deep learning models can efficiently screen for candidate linear epitopes, integrating heterogeneous data across immunology, structural biology, and biophysics remains highly complex (95). The dynamic nature of *in vivo* immune responses, combined with the intricacies of peptide-MHC binding and T-cell activation, limit the accuracy of vaccine efficacy predictions (96). Critically, high performance metrics (e.g., AUC for binding prediction) often fail to translate to generalizable, biologically relevant epitope identification, as they can be inflated by artifacts including sequence similarity between training and test sets, non-independent negative samples, HLA allele imbalance, data leakage, or assay-specific biases. Rigorous validation protocols are required to mitigate these biases, including sequence-identity-aware splitting, pathogen-held-out evaluation, temporal validation, HLA-allele-held-out testing, external validation, calibration, uncertainty estimation, precision-recall analysis, and benchmarking against simple baseline models. The underrepresentation of rare HLA alleles and non-European populations in commonly used training datasets further constrains model generalizability (97). Dependence on limited or skewed training data also impedes AI’s capacity to predict cross-reactivity and off-target effects, hindering development of safe and effective vaccines. Enhanced cross-disciplinary collaboration among immunologists, structural biologists, and data scientists is essential to address these challenges.

5. Rational design–functional immune response gap A profound bottleneck in AI-assisted linear epitope vaccine development lies in bridging the gap between in silico rational design and functional *in vivo* immune responses (98). Current AI models excel at predicting static protein structures but fail to capture the dynamic conformations of B-cell epitopes on native pathogen surfaces (99). As a result, designed immunogens (e.g., stabilized cyclic peptides) may adopt artificial conformations that are not recognized by neutralizing antibodies (100), creating a critical failure mode: even when AI optimizes antibody affinity for synthetic immunogens, the resulting antibodies often fail to recognize native viral proteins, with no detectable neutralization activity even at concentrations exceeding 100 μg/mL (101). Documented translational failures underscore this gap. For example, a 2023 study reported that computationally optimized HIV-1 vaccine peptide epitopes with high predicted MHC-binding affinity failed to elicit neutralizing antibodies in rabbit immunizations, despite strong *in vitro* T-cell responses (91). Similarly, a multi-epitope SARS-CoV-2 vaccine designed via immunoinformatics showed robust predicted CD8^+^ T-cell epitope coverage across global populations, but ELISpot assays in HLA-transgenic mice revealed that only 40% of predicted epitopes actually induced detectable IFN-γ responses (70). These examples demonstrate that state-of-the-art in silico predictions cannot substitute for experimental validation, as the biological complexity of antigen processing, presentation, and TCR recognition introduces failure modes unaccounted for by current computational frameworks. To address this bottleneck, future work should integrate molecular dynamics simulation data, multi-conformation cryo-EM structures, and *in vitro* neutralization datasets to train next-generation AI models that can holistically map the relationships between immunogen conformation, antibody response, and protective efficacy.

### 5.2 Additional critical considerations

Safety and Manufacturing Despite being widely regarded as inherently safe due to their defined chemical composition, the safety of peptide vaccines cannot be assumed *a priori*. Potential risks include molecular mimicry-driven autoimmunity, off-target T-cell recognition, cross-reactivity with human proteins, cytokine-mediated toxicity, anti-carrier immunity, allergic reactions, and adjuvant-related toxicity. HLA-restricted adverse responses may also arise due to global HLA allele diversity. Manufacturing-related risks must also be addressed, including poor peptide solubility, aggregation, oxidation, deamidation, inconsistent chemical purity, challenges in long-peptide synthesis, batch-to-batch variability, formulation instability, inadequate analytical characterization, sterility failures, endotoxin contamination, and suboptimal storage conditions. AI-driven toxicity prediction should be treated as a preliminary prioritization tool, not a replacement for rigorous experimental toxicology and clinical safety evaluation (102).

Population Coverage and Equity While HLA allele frequency is often cited as a key determinant of population coverage, this metric is far more nuanced than predicted binding to a limited set of common alleles suggests. A broadly protective, globally applicable linear epitope vaccine requires consideration of multiple interconnected factors: HLA haplotype diversity, linkage disequilibrium, combined class I and II allele coverage, regional HLA variation, database sampling bias, antigen processing efficiency, immunodominance competition, and discrepancies between predicted and observed *in vivo* response rates. Host factors including age, history of prior infection or vaccination, immunosuppressive status, and TCR repertoire diversity also modulate individual vaccine responsiveness. These considerations directly link AI-driven epitope selection to the broader goals of precision vaccinology and global health equity (103).

### 5.3 Future development trends

The trajectory of linear epitope subunit vaccine development is primed for innovation driven by two core paradigms: AI-assisted design and precision delivery. The advent of AI has significantly advanced the design of these vaccines, streamlining antigen optimization, screening pipelines, and clinical trial design (97). The integration of AI with immunoinformatics is reshaping traditional research paradigms, as exemplified by the use of AlphaFold2 for protein structure prediction coupled with graph neural networks (GNNs), which establishes an iterative feedback cycle between virtual design and experimental validation (104).

Progress in delivery technologies is also advancing rapidly, with biomimetic NPs showing promise in enhancing CD8^+^ T cell activation via targeted modulation of dendritic cell (DC) antigen presentation pathways (105). Personalized vaccine development is entering a new era of dynamic precision, powered by technologies such as single-cell multi-omics integrated with liquid biopsy (106) and microfluidic organ-on-chip-derived digital twin models, which claim to predict vaccine efficacy within 72 hours (107). However, these assertions—including claims that digital twin models can predict vaccine efficacy within 72 hours and that implantable vaccine micro-factories will enable real-time immune monitoring—remain largely speculative, and should be regarded as prospective research hypotheses rather than proven, clinically validated capabilities. Future implantable vaccine micro-factories could enable real-time immune monitoring and regulation, paving the way for a new era of precision immune engineering.

AI is revolutionizing the prediction of immune outcomes for linear epitope-based vaccines, particularly in deciphering the complex interplay of the immune system (95). While this promise is considerable, it is critical to recognize well-documented limitations: several AI-predicted epitope vaccines have failed to confer protective immunity in preclinical models, highlighting that current AI-driven approaches lack the reliability to forecast *in vivo* efficacy (91). The optimism surrounding AI-driven design must be tempered by these demonstrated constraints, particularly the failure of existing models to incorporate antigen processing dynamics, immunodominance hierarchies, and the multifaceted crosstalk between innate and adaptive immune responses that ultimately determines vaccine success. Traditional vaccine development has historically focused narrowly on epitope prediction; however, AI applications mandate a holistic, integrative framework that encompasses multiple hierarchical levels of immune regulation—including immune recognition, activation, and effector responses—rather than focusing exclusively on epitope structures (98). Achieving systematic prediction of vaccine-induced immune outcomes by integrating multidimensional parameters, such as HLA polymorphism, T-cell receptor (TCR) repertoire diversity, and cytokine network dynamics, represents a critical frontier for future research.

In virtual immune prediction, AI holds immense promise. With advances in computational capacity and algorithmic optimization, AI can enhance predictive accuracy for both conformational and linear epitopes by integrating datasets from structural immunology and systems biology (98). This cross-disciplinary integration enables the identification of highly conserved, immunogenic pathogen targets. For immune escape prediction, deep learning models can track pathogen evolutionary trajectories in real time, providing a robust theoretical foundation for the design of broad-spectrum vaccines and optimization of immunization regimens. Leveraging single-cell sequencing and multi-omics data, AI can be used to develop personalized immune response prediction models, driving the advancement of precision vaccinology. Through large-scale molecular dynamics simulations and virtual immune screening, AI technologies can markedly accelerate the optimization of vaccine candidates, dramatically reducing both the time and cost associated with preclinical research (108). Amid the rising threat of emerging infectious diseases and persistent global vaccine inequity, these AI-powered technologies will play a pivotal role in bolstering the resilience of public health systems.

Multiple comprehensive reviews on AI-assisted vaccine design have been published between 2025 and 2026, spanning topics from epitope prediction algorithms to nanoparticle delivery systems. The unique value of the present review lies in its dedicated focus on linear epitope-based subunit vaccines, which integrates computational prediction, chemical modification, delivery technologies, and clinical translation into a cohesive analytical framework. Instead of providing an exhaustive inventory of all available tools, we critically benchmark leading prediction platforms against published performance data, characterize common failure modes, and highlight the translational gap between in silico predictions and functional immune outcomes. This review is not intended as a comprehensive tool catalog, but rather as a practical reference for researchers seeking to delineate both the current capabilities and well-documented limitations of AI-driven linear epitope vaccine design.

## 6 Conclusion

Research on linear epitope subunit vaccines has advanced significantly in the fields of infectious disease prevention and cancer immunotherapy, offering inherent advantages including high specificity, optimized immunogenicity, low toxicity, and well-standardized manufacturing processes. The integration of computer-aided design technologies with bioinformatics tools has greatly improved vaccine design efficiency, accelerating target screening and validation processes. Nevertheless, several persistent challenges remain, particularly those related to immune tolerance, which vary across clinical indications: breaking immune tolerance for cancer therapy, inducing tolerance for autoimmune disease intervention, and avoiding tolerance induction for infectious disease vaccines. Additional hurdles include the instability of free linear peptides and epitope escape driven by pathogen mutations.

Of particular importance is bridging the gap between computational prediction and biological validation: most AI-identified epitopes have not yet been prospectively validated in preclinical animal models or human clinical trials, and multiple high-profile predictions have failed to confer protective immunity *in vivo*. Future research should prioritize multidisciplinary collaboration to drive innovation in AI-optimized epitope-carrier design, develop novel adjuvant systems to enhance immune activation, and explore neoantigen-based personalized vaccine strategies. These efforts are essential for overcoming existing technological bottlenecks and advancing the clinical translation of linear epitope subunit vaccines for effective disease prevention and treatment.
